# Multichannel bridges and NSC synergize to enhance axon regeneration, myelination, synaptic reconnection, and recovery after SCI

**DOI:** 10.21203/rs.3.rs-3044426/v1

**Published:** 2023-07-19

**Authors:** Usha Nekanti, Pooja Sakthivel, Atena Zahedi, Dana A. Creasman, Rebecca A. Nishi, Courtney M. Dumont, Katja M. Piltti, Glenn L. Guardamondo, Norbert Hernandez, Xingyuan Chen, Hui Song, Xiaoxiao Lin, Joshua Martinez, Lillian On, Anita Lakatos, Kiran Pawar, Brian T. David, Zhiling Guo, Stephanie K. Seidlits, Xiangmin Xu, Lonnie D. Shea, Brian J. Cummings, Aileen J. Anderson

**Affiliations:** 1. Sue and Bill Gross Stem Cell Research Center, University of California, Irvine, CA, USA; 2. Department of Anatomy and Neurobiology, University of California, Irvine, CA, USA; 3. Department of Physical Medicine and Rehabilitation, University of California, Irvine, CA, USA; 4. Institute for Memory Impairments & Neurological Disorder, University of California Irvine, CA, USA; 5. Department of Medicine & Susan Samueli Integrative Health Institute, University of California, Irvine, CA, USA; 6. Department of Biomedical Engineering, University of Michigan, Ann Arbor, MI, USA; 7. Department of Biomedical Engineering, The University of Texas at Austin, TX, USA; 8. Department of Medicine, University of British Columbia – Vancouver, Canada; 9. Department of Neurosurgery, Rush University Medical Center, Chicago, Illinois, USA

## Abstract

Regeneration in the injured spinal cord is limited by physical and chemical barriers. Acute implantation of a multichannel poly(lactide-co-glycolide) (PLG) bridge mechanically stabilizes the injury, modulates inflammation, and provides a permissive environment for rapid cellularization and robust axonal regrowth through this otherwise inhibitory milieu. However, without additional intervention, regenerated axons remain largely unmyelinated (<10%), limiting functional repair. While transplanted human neural stem cells (hNSC) myelinate axons after spinal cord injury (SCI), hNSC fate is highly influenced by the SCI inflammatory microenvironment, also limiting functional repair. Accordingly, we investigated the combination of PLG scaffold bridges with hNSC to improve histological and functional outcome after SCI. In vitro, hNSC culture on a PLG scaffold increased oligodendroglial lineage selection after inflammatory challenge. In vivo, acute PLG bridge implantation followed by chronic hNSC transplantation demonstrated a robust capacity of donor human cells to migrate into PLG bridge channels along regenerating axons and integrate into the host spinal cord as myelinating oligodendrocytes and synaptically integrated neurons. Axons that regenerated through the PLG bridge formed synaptic circuits that connected ipsilateral forelimb muscle to contralateral motor cortex. hNSC transplantation significantly enhanced the total number of regenerating and myelinated axons identified within the PLG bridge. Finally, the combination of acute bridge implantation and hNSC transplantation exhibited robust improvement in locomotor recovery vs. control and hNSC transplant alone. These data identify a successful novel strategy to enhance neurorepair through a temporally layered approach using acute bridge implantation and chronic cell transplantation to spare tissue, promote regeneration, and maximize the function of new axonal connections.

## INTRODUCTION

Penetrating spinal cord injury (SCI), which accounts for a significantly smaller proportion of injuries, offers little opportunity for acute neuroprotection, and may require true axonal regeneration for repair. Axons in the spinal cord do retain some growth capacity; however, this potential is limited by the presence of inhibitory scar tissue, growth-inhibitory molecules, and myelin-associated glycoprotein molecules [1–5]. Decades of research on SCI regeneration have made it clear that this multifactorial problem is likely to require a combinatorial solution [6–10].

Implanted biomaterial scaffolds are one path by which to attempt to restore function after SCI. Functionalization of an injectable hydrogel matrix with an enzyme to degrade CSPGs with injection into a SCI cavity has been shown to enable serotonergic axon growth through the biomaterial, with exit into the caudal spared parenchyma and evidence of locomotor recovery; however, hydrogel alone showed neither axon growth or function [11]. Similarly, acute implantation of a 3D-printed scaffold pre-seeded with neural cells resulted in improvement of joint movements; locomotor improvement in this model was proposed to result from formation of a bridge circuit within the pre-seeded scaffold, in part because implantation of the scaffold alone did not yield recovery [12]. Finally, acute injection of bioactive scaffolds have been shown to promote regeneration through the site of a severe SCI in mice with recovery of function; however, locomotor improvement is evident within one week, which is likely to reflect tissue sparing and not regeneration [13]. In an alternative approach, we have shown that implantation of an empty multi-lumen poly (lactide-co-glycolide) (PLG) bridge enables corticospinal tract (CST) axons to regenerate through the full length of the bridge and cross into the spared parenchyma below the implantation site, producing functional locomotor recovery on a ladder task beginning 10 weeks later, a timeline consistent with a mechanism that requires axon regeneration as opposed to tissue sparing [14].

Whether axons that regenerate into implanted biomaterials become remyelinated has not received a great deal of attention. In the PLG bridge, for example, only 5–10% of regenerated axons are associated with oligodendrocyte derived myelin [15–17]. While myelination may not be required for spontaneous recovery after contusion SCI [18], it is an essential factor for efficient transmission of signals from the motor cortex to the spinal cord in the healthy CNS; indeed, 86% of the rodent CST and 99% of the normal human CST is myelinated [19, 20]. The impact of myelination of regenerated axons on function is unknown. Endogenous CNS stem cells become activated in response to SCI [21, 22], but the capacity of these cells for remyelination is limited by both time and distance from the lesion epicenter [23, 24], with steady declines in these populations by 4 weeks post-injury [21, 24–26]. This suggests that the endogenous progenitor response to the injury goes quiescent before axonal regeneration is achieved through the PLG bridge, identifying a temporal mismatch. We sought to address this gap by investigating acute bridge implantation paired with chronic hNSC transplantation. To accomplish this, rather than implanting a seeded bridge as other studies have tested, we delayed hNSC delivery by transplanting into the spared parenchyma adjacent to the bridge 30 days later, analyzing migration of donor cells into axon containing bridge channels and recovery of function.

**Figure 1:**
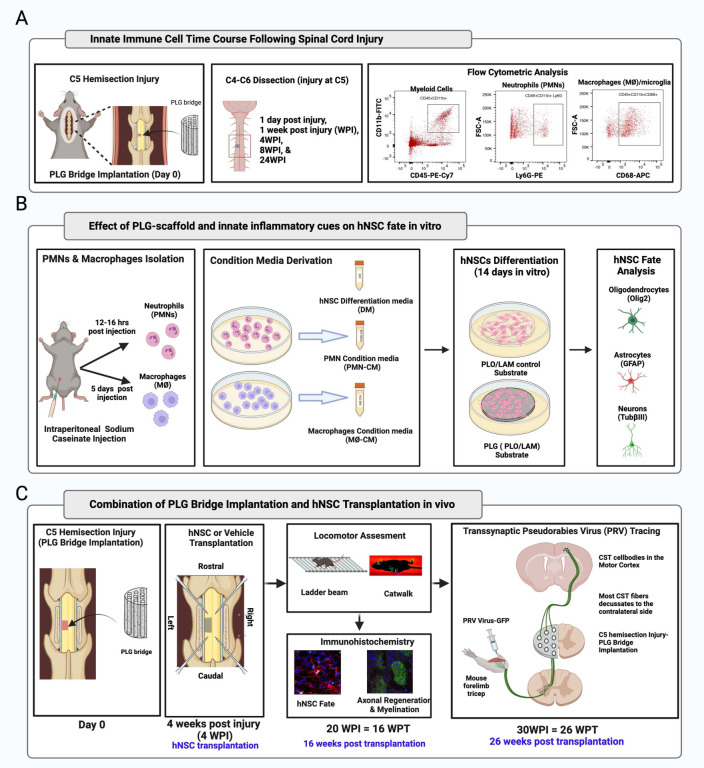
Experimental design schematic. (A) Quantitative innate immune cell profiling in the presence of PLG bridge following SCI. Schematic shows C5 hemisection injury and implantation of PLG bridge at the lesion site. C4 to C6 spinal cord segments (injury at C5) were dissected at the following time points post-injury, 1 day post-injury (DPI), 1 week post-injury (WPI), 4 WPI, 8 WPI, and 24 WPI. Dissected spinal cord tissues were processed for flow cytometric analysis; a representative flow cytometry plot shows gating for the total myeloid population (CD45+ CD11b+). The myeloid population was further gated for Ly6G^+^ neutrophils (PMN) and CD68^+^ macrophage (MØ)/microglia subpopulations, and innate immune cell proportions profiled in C57BL/6 (Fig. 2) and Rag1 (**Supplemental Fig. 1**) mice. **(B)** *In vitro* analysis of PLG-scaffold and innate immune cues on hNSC cell fate. PMN and MØ were isolated from the peritoneal cavity of Rag1 immunodeficient mice stimulated with 12% sodium caseinate (i.p.). PMN and MØ were subsequently cultured in hNSC differentiation medium (DM), and respective conditioned media (PMN-CM, MØ-CM) was collected. hNSC were differentiated on PLO/LAM (control substrate) and PLG scaffold in the presence of DM, PMN-CM, and MØ-CM. hNSC fate was quantified using Imaris software following immunocytochemistry. **(C)** Timeline for the combinatorial approach of PLG bridge implantation and hNSC transplantation in Rag1 immunodeficient mice. Mice received C5 left hemisection injury and immediate PLG bridge implantation and dorsal surface covered with gelfoam; SCI control mice received only gelfoam implantation. 4WPI, mice received either vehicle injection or hNSC transplantation into the spared tissue parenchyma at two sites both rostral and caudal to the lesion site. Mice were randomly distributed into SCI control vs. PLG bridge groups at the time of initial surgery, and into the vehicle vs. hNSC groups on the day of transplantation. hNSC fate and distribution, host axonal regeneration, myelination status, and locomotor recovery were analyzed at 16 weeks post-transplantation (WPT). Finally, transsynaptic PRV retrograde tracing was performed at 26 WPT (30 WPI) to investigate synaptic connectivity of regenerated axons.

Critically, transplanted hNSC migrating into the PLG bridge will encounter an altered immune microenvironment [16, 27, 28]. We have shown that immune cells influence the fate, migration, functional integration, and efficacy of NSC transplanted after SCI [29–33]. We therefore first tested the effect of the PLG bridge on the time course of the innate immune response after SCI *in vivo* (Fig. 1A). We further tested the effect of interaction with the PLG biomaterial on hNSC fate *in vitro*, alone or in combination with immune factors (Fig. 1B). Finally, we evaluated the effect of combining PLG bridge implantation and hNSC transplantation *in vivo* (Fig 1C), analyzing hNSC fate, CST regeneration and myelination, and locomotor recovery. Our data identify a novel strategy to enhance neurorepair through a temporally layered approach using acute bridge implantation and chronic hNSC transplantation to spare tissue, promote regeneration, and maximize the function of new axonal connections.

## RESULTS

### PLG bridge implantation modulates the cellular immune response in vivo by prolonging the macrophage/microglial response.

We have previously shown that SCI is associated with a multiphasic immune cell response and SCI inflammatory microenvironment modulates hNSC fate, migration, and potential for repair after *in vivo* transplantation [31, 32, 34]. In parallel, we have reported that macrophages migrate to and phagocytose implanted biodegradable PLG bridges [14, 28]. Critically, no deleterious effects of PLG are observed *in vitro,* or *in vivo* after bridge implantation [14, 16, 17, 28, 35–37], and lactate, a PLG biodegradation product, polarizes immune cells towards tolerogenic phenotypes [38, 39]. Accordingly, we first tested how PLG bridge implantation modulates the innate immune cell response within the bridge and surrounding spared spinal cord tissue at different time points following injury. We utilized a quantitative flow cytometry-based method to characterize this multiphasic response in a hemisection injury model for PLG bridge and SCI control animals (Fig. 1A).

**Figure 2:**
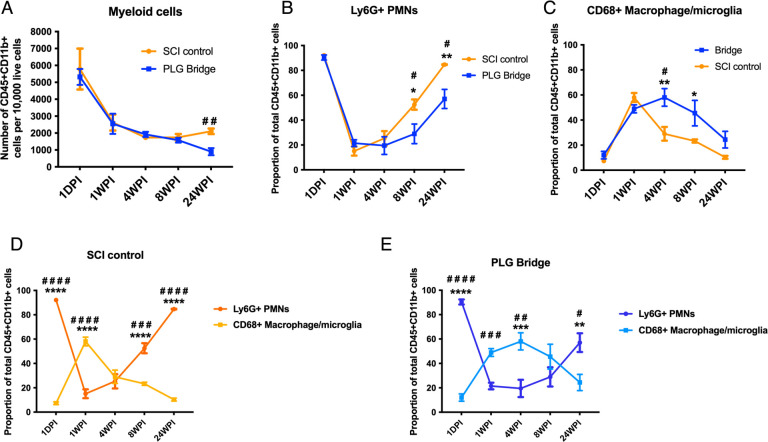
PLG bridge implantation modulates innate immune cell response and time course in the injured C57B/6 mouse spinal cord. **(A)** The total myeloid cell population infiltration in the SCI control (orange) group and PLG (blue) groups over time. **(B-C)** Proportions of PMN (B) and MØ/microglia (C) subpopulations were shown over time. **(D)** Comparison of the Ly6G^+^ PMN (dark orange circle line) and MØ/microglia (light orange square line) ratios in the SCI control group. **(E)** Comparison of the Ly6G^+^ PMN (dark blue circle line) and MØ/microglia (light blue square line) ratios in the PLG group. Comparisons showing * are using 2-way ANOVA, followed by Sidak test (****p ≤0.0001, ***P≤0.001, **P≤0.01, *P≤0.05). Comparisons showing ^#^ are using unpaired t-tests at each time point (^####^p ≤0.0001, ^###^P≤0.001, ^##^P≤0.01, ^#^P≤0.05). Graphs represent Mean ± SEM; N=4–5 mice/group

PLG bridge and SCI control groups behaved similarly until 24 weeks post-injury (WPI), where the number of myeloid cells were significantly reduced in PLG vs. SCI control mice at 24 WPI (Fig. 2A). We next investigated the proportion of Ly6G^+^ polymorphonuclear leukocytes (PMN) and CD68^+^ macrophages (MØ)/microglia in the total myeloid population. As demonstrated previously after contusion SCI in rodents [34], the epicenter environment was dominated by PMN in both SCI control and PLG groups at 1 day postinjury (DPI) (Fig. 2B). PMNs were dramatically reduced at 1 and 4 WPI. Similarly, there a delayed reemergence of the PMN population observed at 8 and 24 WPI. This second phase of PMN infiltration was still present, but significantly reduced, in PLG bridge vs. SCI control mice (Fig. 2B). As expected from previous analysis in contusion SCI, MØ/microglia populations were sparsely detected at 1 DPI (Fig. 2C) and exhibited a later peak in response to injury. However, while the PLG bridge and SCI control groups were similar at 1 WPI, the MØ/microglia subpopulation peak was significantly extended in PLG bridge vs. SCI control mice (Fig. 2C). The observation of an extended phase of macrophage/microglia recruitment after PLG bridge implantation is consistent with the known role for the MØ population in phagocytosis and biodegradation of PLG [40]. Analysis of PMN and MØ/microglia populations as a ratio is a useful way to visualize the dynamic balance of these immune populations within the spinal cord [34], highlighting an enhanced PMN response in the SCI control group (Fig. 2D) and an extended phase of MØ /microglia recruitment in the PLG bridge group (Fig. 2E). Thus, the subacute/chronic injury time point of 4–24 WPI represents a window in which the PLG bridge modulates the cellular inflammatory response.

### hNSC culture on PLG substrate in the presence or absence of immune factors enhances oligodendroglial and suppresses astroglial lineage selection.

Given the impact of PLG bridge on innate immune cell time course, we next assessed the effect of PLG bridge on hNSC fate in the absence or presence of immune cues. Studies have shown that the physical, mechanical, and biochemical properties of biomaterial scaffolds can influence NSC differentiation, neurite extension, and cell morphology, as well as change actin structure and focal adhesion molecules [41–43]. The potential for multipotent donor hNSC to elicit repair after transplantation is strongly linked to fate selection [29, 31, 32, 44]. Accordingly, we tested the effect of PLG vs. poly-L-ornithine /laminin (PLO/LAM) control substrate on hNSC fate (Fig. 1B). Additionally, innate immune cells and their secreted components within the microenvironment have been shown to influence the outcome of hNSC transplantation, resulting in changes in fate, migration, and efficacy [30–32]. We therefore also tested the effect of PLG on hNSC fate in the presence of innate immune cues, utilizing PMN and MØ conditioned media (CM) (Fig. 1B & Supplemental Fig. 2A-C).

**Figure 3:**
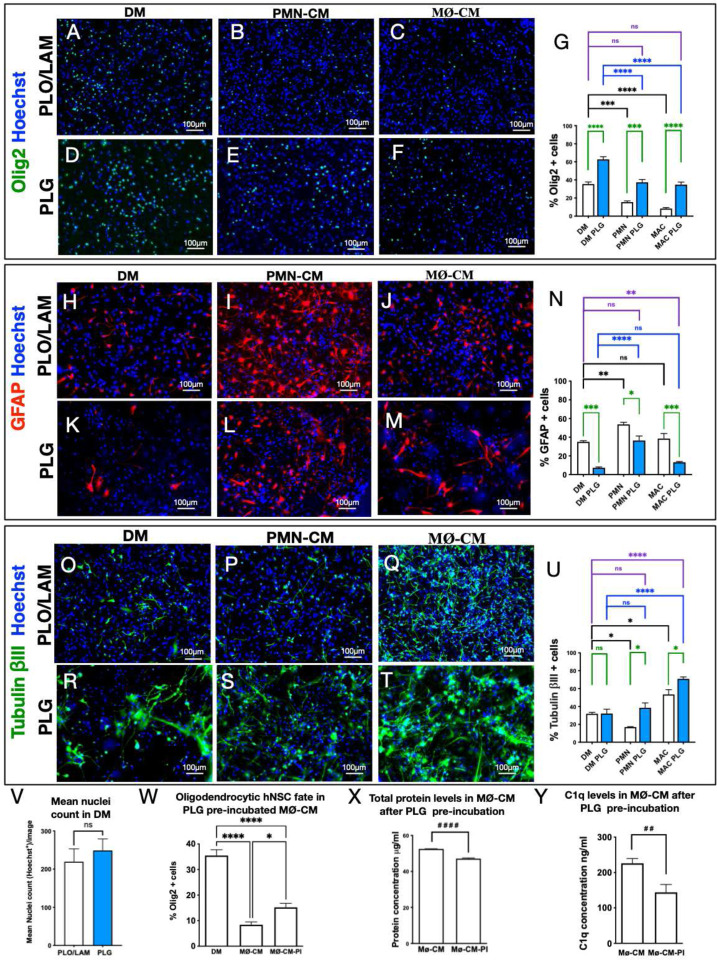
PLG substrate alters hNSC fate at baseline and in response to immune cues in vitro, enhancing oligodendroglial and suppressing astroglial fate. **(A-U)** Representative images and quantification of hNSC differentiation profile on either PLO/LAM or PLG substrate with differentiation medium (DM), PMN conditioned medium (PMN-CM), and macrophage conditioned medium (MØ-CM. Quantitative comparisons were performed for the following markers. (**A-G**) Oligodendrocytic fate. (**H-N**) Astrocytic fate. (**O-U**) Neuronal fate. Statistical analysis One-way ANOVA, followed by Tukey post hoc tests (****p ≤0.0001, ***P≤0.001, **P≤0.01, and *P≤0.05). Graphs show Mean ± SEM; N = 3–4 biological replicates/condition. Black lines: DM control vs. PMN-CM and MØ-CM on PLO/LAM substrate. Blue lines: DM control vs. PMN-CM and MØ-CM on PLG substrate. Green lines: PLO/LAM vs. PLG substrate. Purple lines: PLG-CM vs. PLO/LAM-DM control. (**V**) Hoechst nuclei counts of hNSC differentiated in the presence of PLG scaffold vs. PLO/LAM control revealed no significant differences by substrate. Graph shows Mean ± SEM; unpaired t-test two-tailed P=0.5404. (**W-Y**) Sequestration of immune cues by PLG was investigated using MØ-CM collected after pre-incubation with PLG scaffold (MØ-CM-PI). (**W**) Preincubation (PI) of MØ-CM with PLG scaffold partially restored hNSC oligodendrocytic fate on PLO/LAM substrate. Comparisons showing * using One-way ANOVA (P<0.0001), followed by Tukey post hoc tests (****p ≤0.0001 and *P≤0.05). (X) MØ-CM total protein levels were significantly reduced by pre-incubation with PLG scaffold, indicating protein binding to the PLG substrate. **(Y)** MØ-CM C1q protein was significantly reduced by pre-incubation with PLG scaffold, indicating binding to the PLG substrate. Groups were compared using unpaired Student’s t-test (^**####**^p ≤0.0001 and ^**##**^P≤0.01), as shown. Graphs show Mean ± SEM; N = 4 biological replicates/condition.

As reported previously, both PMN-CM and MØ-CM suppressed oligodendroglial fate on PLO/LAM (Figs. 3A–C, Fig. 3G) [32]. While hNSC cultured on PLG scaffold exhibited a similar effect, PLG drove a highly significant increase in oligodendroglial fate under baseline conditions, and rescued oligodendroglial fate under PMN-CM and MØ-CM conditions Figs. 3D–F, Fig. 3G). Consistent with our previous observation, PMN-CM enhanced astroglial fate and suppressed neuronal fate, while MØ-CM had no effect on astroglial fate and enhanced neuronal fate [32]. hNSC cultured on PLG substrate modulated these effects, reducing astroglial fate at baseline and in response to PMN-CM and MØ-CM, and enhancing neuronal fate in response to PMN-CM and MØ-CM (Fig. 3H–U). However, PLG showed no significant effect vs. PLO/LAM on the total number of cells after 14 days *in vitro* (DIV) in differentiation media (DM) via nuclei (Fig. 3V). Collectively, these data indicate that PLG substrate alters hNSC fate at baseline and in response to immune cues.

The enhancement of baseline oligodendroglial fate, and suppression of astroglial fate, by culture on PLG scaffold vs. PLO/LAM indicates that direct signaling to hNSC via cell-substrate contact plays an important role in modulation of differentiation. PLG could also sequester soluble molecules contained within the differentiation media as well as in CM derived from these immune cell populations. We tested this possibility via a pre-incubation (PI) paradigm, focusing on oligodendroglial fate in response to MØ-CM as a read out. Treatment of hNSC cultured on PLO/LAM with MØ-CM collected after pre-incubation with PLG scaffold (MØ-CM-PI) produced a partial restoration of oligodendroglial fate (Fig. 3W). In parallel, quantification of protein concentration in MØ-CM and MØ-CM-PI showed a reduction in total protein concentration (Fig. 1X). We have previously demonstrated that complement component C1q is a key mediator of the effects of PMN-CM and MØ-CM on hNSC fate, and that C1q neutralizing antibodies reverse the effect of CM on hNSC fate [32]. We therefore further tested for sequestration of soluble molecules by ELISA analysis of C1q protein levels present in MØ-CM cultures on PLG substrate, identifying an ~40% reduction in available C1q (Fig. 3Y). Together, these data suggest that the PLG scaffold supports adventitious modulation of donor stem cell fate via modulation of the bioavailability of immune cell or hNSC secreted factors in addition to the physical and mechanical properties of the biomaterial substrate [41–43, 45–47].

### PLG bridge implantation supports robust hNSC engraftment in the spared tissue parenchyma rostral and caudal to the lesion site.

We selected the timing of chronic hNSC transplantation based on the following considerations. First, axons enter and extend into the channels of the PLG bridge by 4WPI. We predicted that these newly formed axons would strongly cue hNSC to migrate and undergo oligodendroglial lineage selection, enabling myelination of axons within the PLG bridge [48, 49]. Second, the SCI epicenter environment is dominated by MØ rather than PMN at 4WPI (Fig. 2). *In vitro* testing showed that, in the presence of MØ-derived cues, hNSC oligodendroglial lineage selection was rescued, astroglial lineage selection was reduced, and neural lineage selection was maximized by PLG scaffold – optimizing potential for repair. Third, chronic transplants are a more clinically relevant time period for delivery of cellular therapies in humans, enabling both improved informed consent and a more medically stable population given the requirement for immunosuppression in an allogeneic transplant setting, minimizing serious adverse effects [50].

We have reported that immunodeficient models lacking T cells are required to enable both xenogeneic and allogeneic CNS engraftment at levels sufficient to analyze sustained effects of donor NSC in the host environment [29, 51]. These models are similar to the translational setting, in which allogeneic stem cell transplantation requires long-term administration of pharmacological immunosuppressants in humans, which similarly target lymphoid cells. Accordingly, these studies utilize Rag1 mice, which lack mature T and B cells but retain a functional innate immune cell response [52]. We first confirmed that the innate immune cell profile in Rag1 mice for the hemisection injury (Supplemental Fig. 1) is similar to the profile observed in C57BL/6 mice (shown in Fig. 2). As expected, Rag1 mice have fewer innate immune cells (CD45^+^CD11b^+^ cells) in comparison to C57BL/6 mice, since they lack T-cells which orchestrate the innate immune response (Fig. 2A, Supplemental Fig. 1A). Importantly however, PMN and MØ/microglia cell proportions closely paralleled that of C57BL/6 mice (Fig. 2D–E, Supplemental Fig.1B-C) after SCI, from 1 DPI through 24 WPI, demonstrating that innate immune responses are, in essence, conserved between these models, which would be predicted to be similar to pharmacological immunosuppression in humans.

**Figure 4:**
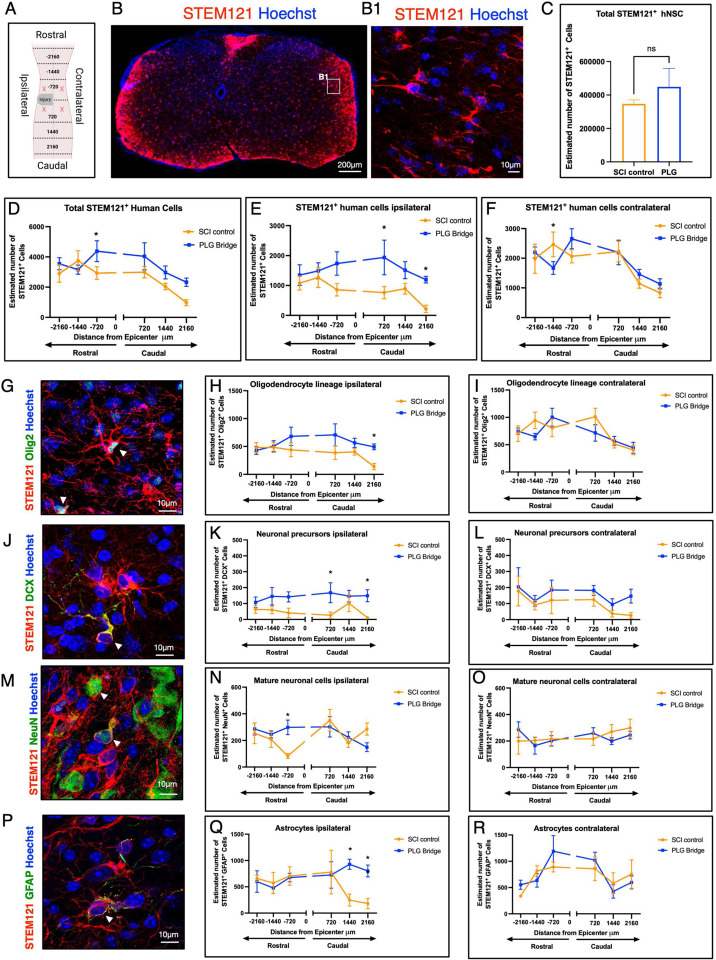
Stereological analysis of hNSC fate and distribution along the spinal cord. **(A)** Schematic of spinal cord injury, showing the lesion epicenter at C5 and indications of coordinates in μm. ‘X’ represents locations of hNSC transplantation at two injection sites rostral and two sites caudal to the lesion epicenter. Ipsilateral refers to the same side as the lesion, and contralateral refers to the side opposite the lesion. Dashed lines represent rostral-caudal axis binning used for histological quantification (720, 1440, 2160 microns from lesion epicenter). **(B)** Representative image of a coronal spinal cord section showing distribution of STEM121^+^ transplanted human hNSC (red) and total cell nuclei (blue); inset (B1) shows higher power (scale bars as indicated). **(C)** Stereological quantification of the total number of STEM121^+^ cells in rostral and caudal regions in PLG and SCI control groups. Graph represents Mean ± SEM; N=3–4 mice/group. Statistical analysis by unpaired Student’s two-tailed t-tests. **(D-F)** Distribution of transplanted STEM121^+^ hNSC in the spinal cord: **(D)** ipsilateral and contralateral combined; **(E)** ipsilateral; and **(F)** contralateral. **(G-R)** Representative images and quantification for cell fate. STEM121^+^/OLIG2^+^ oligodendrocytes: **(G)** Immunostaining; **(H)** ipsilateral; **(I)** contralateral. STEM121^+^/DCX^+^ neuronal precursors: **(J)** Immunostaining; **(K)** ipsilateral; **(L)** contralateral. STEM121^+^/NeuN^+^ mature neurons: **(M)** Immunostaining; **(N)** ipsilateral; **(O)** contralateral. STEM121^+^/GFAP^+^ astrocytes: **(P)** Immunostaining; **(Q)** ipsilateral; **(R)** contralateral. Statistical analysis by unpaired Student’s two-tailed t-tests (*p≤0.05, **p≤0.005; Mean ± SME; N=5–6/group).

**Figure 5:**
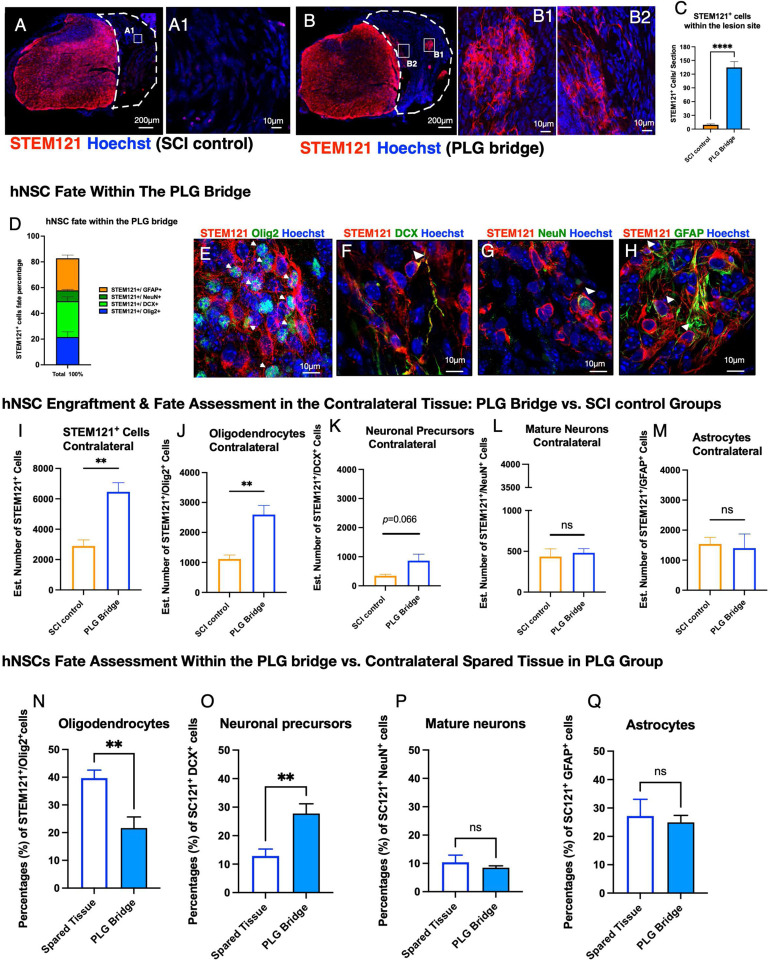
PLG bridge implantation creates a permissive environment for hNSC engraftment and enhances oligodendroglial and neuronal fate. **(A)** No hNSC engraftment was observed in the spinal cord lesion site for the SCI control group; inset **(A1)** shows higher power (scale bars as indicated). **(B)** Numerous hNSC migrated into biomaterial channels in the PLG group; insets **(B1 and B2)** show higher power (scale bars as indicated). White dashed lines outline the lesion site. **(C)** Quantification of total STEM121^+^ hNSC comparing SCI control and PLG groups. Because donor cell distribution was not homogenous within the control and PLG bridge, we performed manual quantification of the total number of cells per 30μm tissue section. **(D)** Quantification of hNSC fate within the PLG bridge. Data represents the percentage of each lineage. Representative images of hNSC fate in the PLG bridge for: **(E)** STEM121^+^/Olig2^+^ oligodendrocytes; **(F)** STEM121^+^/DCX^+^ neuronal precursors; **(G)** STEM121^+^/NeuN^+^ mature neurons; and **(H)** STEM121^+^/GFAP^+^ astrocytes. **(I)** Quantification of total STEM121^+^ hNSC in the spared tissue contralateral to the lesion/bridge implantation site. **(J-M)** Quantification of hNSC fate in the contralateral side comparing control and PLG groups. **(N-Q)** Comparison of proportional hNSC fate within the bridge (data from D) to hNSC fate within the spared contralateral side of the same section (data from J-M). Statistical analysis by unpaired two-tailed t-tests (**p≤0.005, ****p≤0.0001; Mean ± SEM; N=5–6/group).

hNSC were transplanted into the spared parenchyma rostral and caudal to the SCI control or PLG bridge implanted lesion site, we therefore first evaluated hNSC engraftment, migration, and fate in these regions at 16 weeks post-transplantation (WPT) (Fig. 1C, Fig. 4). Spinal cord transverse sections were aligned relative to the lesion epicenter and the distribution of transplanted hNSC at defined section distances was analyzed using unbiased stereology (see Methods, Fig. 4A). Coronal sections were immunostained for STEM121 (human stem cell marker) to reveal the distribution of transplanted hNSC (Fig. 4B–B1). Quantification in Fig 4 excludes the site of implantation since distribution within this region is non-uniform and not accessible by stereology; these data are quantified separately in Fig 5.

Total hNSC engraftment within the rostral and caudal spared tissue segments in PLG vs. SCI control groups was comparable (Fig. 4C–D), however, small but significant increases in STEM121+ hNSC as well as hNSC tri-lineage cell fate markers (Olig2+ oligodendrocytes, DCX+ or NeuN+ neurons, and GFAP+ astrocytes) were detected in the spared tissue ipsilateral to the SCI and site of bridge implantation (Fig. E-R). No consistent trends were observed for any of these human cell makers contralaterally. Overall, our results suggest that there is a selective ipsilateral increase in the number of transplanted donor hNSC in the PLG group, and that the combination of PLG scaffold implantation with hNSC transplantation has a small effect on modulation of cell fate within the spared SCI tissue.

### hNSC preferentially localize within PLG bridge channels and exhibit tri-lineage differentiation.

We next determined whether donor hNSC were competent to migrate into the SCI control or PLG bridge implanted lesion site (Fig. 5A and B). Few human cells were detected in the lesion site of the SCI control group (mean = 9 STEM121^+^ cells/section; Figs. 5A, A1 and C), but this was dramatically increased in the PLG group (mean = 145 STEM121^+^ cells/section; Figs. 5B, B1–2 and C). There were too few engrafted human cells for fate quantification in the SCI control group, however, STEM121^+^ hNSC in the PLG implanted group exhibited tri-lineage differentiation by 16 WPT. Cell fate analysis of STEM121^+^ cells within the lesion site identified 21.6% Olig2^+^ cells (Figs. 5D and E), 27.7% DCX^+^ cells (Figs. 5D and F), 8.45% NeuN^+^ (Figs. 5D and G) cells, and 24.9% GFAP^+^ cells (Figs. 5D and H). 17.19% of STEM121^+^ cells were unlabeled for the tested markers (Fig. 5D). hNSC were highly localized within the channels of the PLG bridge adjacent to regenerated axons (Fig. 5B, B1–2), while other cell types (e.g. fibroblasts, macrophages, Schwann cells, oligodendrocytes, and endothelial cells) localized to the porous bridge structure [28] (Fig. 5B). These data demonstrate that hNSC in the bridge differentiate into all three neural lineages, and that hNSC migrate into the PLG bridge along the same channels in which axons are localized.

### Both total hNSC engraftment and the number of hNSC-derived oligodendrocytes are increased in the contralateral spared tissue in PLG bridge vs. SCI control mice.

We have previously shown that PLG bridge implantation stabilizes adjacent spinal cord tissues [14]. We sought to test whether hNSC that engrafted in the spared tissue directly contralateral to the site of PLG bridge implantation vs. SCI control exhibited differences engraftment or fate. The number of total engrafted STEM121^+^ hNSC was significantly increased in the PLG group compared to the SCI control (Fig. 5I), as was the number of STEM121^+^/Olig2^+^ cells (Fig. 5J). While there was a parallel trend for an increase in the number of immature STEM121^+^/DCX^+^ cells (*p-value* =0.06, Fig. 5K), this was not observed for either mature STEM121^+^/NeuN^+^ cells (Fig. 5L) or STEM121^+^/GFAP^+^ hNSC (Fig. 5M). Critically, the proportion of hNSC adopting different lineages was not significantly different between the PLG bridge vs. SCI control groups (data not shown). These data suggest that the principal effect of the PLG bridge was on overall survival/engraftment of hNSC in the spared tissue, which is consistent with the stabilization of this region in the acute period after injury. This difference in engraftment cannot be ascribed to an increased area/volume but may rather reflect a change in the molecular microenvironment and/or mechanostability of this region.

### hNSC within PLG bridge channels exhibit altered fate vs. contralateral spared tissue in SCI epicenter sections.

In previous studies using contusion SCI models, we have shown that hNSC that are localized adjacent to the SCI epicenter are directed towards the astroglial lineage, but that these cells do not migrate into the SCI epicenter per se [29, 31, 44]. As described above, hNSC that migrated along the PLG channels retained tri-lineage potential. Here, we asked whether the fate of hNSC that migrated into the PLG bridge and inflammatory environment of the SCI epicenter was similar to or different from hNSC that engrafted in contralateral spared tissue in the same tissue sections. While hNSC that migrated into the PLG bridge retained the capacity to differentiate along the oligodendroglial lineage (STEM121^+^/Olig2^+^; Fig. 5N), they exhibited increased neuronal lineage selection (STEM121^+^/DCX^+^; Fig. 5O). In contrast, mature neuron (STEM121^+^/NeuN^+^) and astroglial (STEM121^+^/GFAP^+^) proportions were unchanged (Fig. 5P–Q). These data are consistent with the observation that the PLG bridge is dominated by macrophages in the chronic post-SCI phase (Fig. 2 and Supplemental Fig. 1), and the influence of PLG and macrophage-derived immune factors on hNSC fate selection (Fig. 3).

### hNSC transplantation enhances axonal regeneration and remyelination in the PLG bridge.

**Figure 6:**
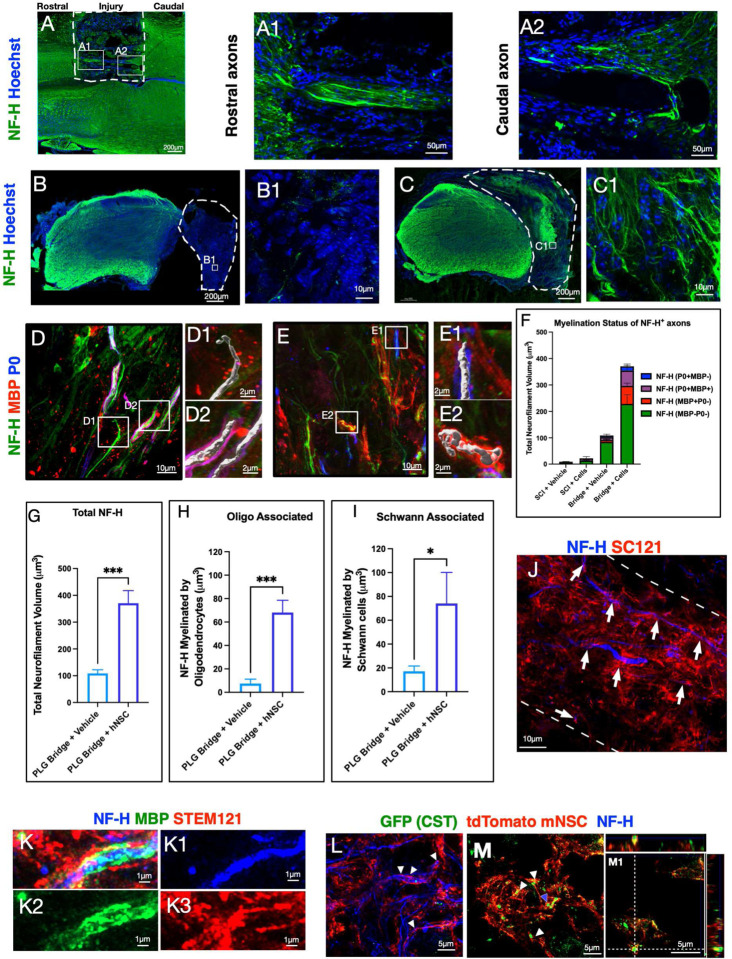
Combination of PLG bridge implantation and hNSC transplantation enhances axonal regeneration and oligodendrocytic myelination. **(A-C)** Immunostaining for total axonal regeneration (NF-H, green) counterstained with a nuclear marker (Hoechst, blue) of SCI control and PLG bridge implanted spinal cord sections. White dashed lines outline the lesion (ipsilateral side) and tissue interface in both control and PLG groups. **(A)** NF-H in a horizontal mouse spinal cord section (C5 left hemisection injury) in which the PLG bridge shows robust axonal regeneration from both the rostral and caudal parenchyma at 6WPI; insets show higher power (**A1-A2**; scale bars as indicated). **(B)** NF-H in a transverse spinal cord section in a SCI control mouse 16 WPT; inset shows higher power (**B1**; scale bars as indicated). **(C)** NF-H in a transverse spinal cord section in a PLG bridge implanted mouse 16 WPT; inset shows higher power (**C1**; scale bars as indicated). **(D-E)** Triple immunostaining for NF-H labeled axons (NF-H; green), oligodendrocyte-derived myelin (MBP; red) and Schwann cell myelin (P0; blue) in PLG bridge implanted spinal cord sections. Boxes indicate regions shown at higher magnification with 3D surface masks representing unmyelinated axons. Examples of unmyelinated axons (**D1** quantified in **F**), Schwann cell myelinated axons (**D2 and E1**; quantified in **F** and **I**, and oligodendrocyte myelinated axons (**E2**, quantified in **F** and **H**). **(F)** Total NF-H+ axon volume and axon proportional myelination status within the PLG bridge. Graph represents Mean ± SEM; N=4–6 mice/group **(G)** Total NF-H volume. **(H)** NF-H volume associated with oligodendrocyte myelin. **(I)** NF-H volume associated with Schwann cell myelin. Graphs represent Mean ± SEM; N=5–6 mice/group. Statistical comparisons conducted using unpaired Student’s t-tests (*p<0.041 and ***p≤0.001) . **(J)** STEM121^+^ processes (red) warping around NF-H axons (blue). **(K)** Colocalization of STEM121 (red) human specific hNSC marker with MBP+ myelin (green) and NF-H+ axons (blue) in the PLG bridge. (**K1-K3**) Individual channel images. **(L)** mT-mNSC exhibit close alignment with regenerated axons in the PLG bridge identified by NF-H^+^ (blue). **(M)** mT-mNSC exhibit close alignment with regenerated CST axons in the PLG bridge identified by CRYM-GFP^+^ (green).

We next investigated axon regeneration and myelination status in these mice by immunostaining SCI control and PLG-implanted spinal cord tissues for neurofilament (NF-H). Consistent with our previous reports describing regeneration of both descending motor and ascending sensory axon [36], NF-H^+^ fibers were readily observed entering PLG bridge channels at both the rostral (Fig. 6A & 6A1) and caudal (Fig. 6A & 6A2) margins by 6 weeks post-injury and bridge implantation.

NF-H^+^ fibers were extremely rare in the lesion site of SCI control animals, even by 20 WPI (16 WPT; Fig. 6B–6B1, Fig. 6F). In contrast, PLG bridge implantation supported robust regeneration of NF-H^+^ axons within the bridge at this timepoint (Fig. 6C–6C1). We quantified the total volume of NF-H+ axons (Fig. 6D–6E), as well as the proportions of axons which were ether unmyelinated (Fig. 6D and D1 inset) or myelinated by MBP^+^P0^−^ oligodendrocyte (Fig. 6E and E2 inset), MBP^+^P0^+^ Schwann cell (Fig. 6D and D2 inset), and/or P0^+^ Schwann cell (Fig. 6E and E1 inset) populations. Analysis of NF-H^+^ axon volume suggests that hNSC transplantation enhanced both regeneration and the myelination status of these axons (Fig. 6F). This effect was substantiated quantitatively by comparison of Imaris volumetric data for PLG bridge alone vs. PLG bridge + hNSC groups (NF-H^+^ regeneration, Fig. 6G; MBP^+^P0^−^ oligodendrocyte-derived myelination, Fig. 6H; MBP^+^P0^+^ plus MBP^−^P0^+^ Schwann cell-derived myelination, Fig. 6I).

Although histological quantification was predominantly performed at 16 WPT, a subset of animals was assessed at 26 WPT. We hypothesized that this additional engraftment time would optimize for the maturation and integration of transplanted hNSC as myelinating cells within the PLG bridge. We therefore tested whether hNSC that entered the bridge channels (white dotted lines in Fig. 6J) were capable of myelinating regenerated axons. Indeed, there was an abundant association of STEM121^+^ hNSC labeling aligned with and in close proximity to NF-H^+^ axons (Fig. 6J, arrowheads). Further, high magnification clearly identifies MBP^+^/STEM121^+^ co-labeling of NF-H^+^ axons (Fig. 6K), indicating that hNSC contributes to myelination of axons that regenerate into PLG bridge channels.

We confirmed this observation and tested whether donor NSC myelinated regenerating descending motor axons using CRYM reporter mice. We have previously employed these mice to specifically visualize CST axons in the spinal cord (Supplemental Fig. 3A), including in the PLG bridge after SCI [14]. Fully functional compact myelin excludes oligodendrocyte cytoplasm, preventing detection of human cells by STEM121 [29]. We therefore derived neural stem cells from mice that ubiquitously express a membrane-targeted fluorescent tandem dimer Tomato (tdTomato) reporter (mT-mNSC) [53] (Supplemental Fig. 3B). This approach allowed mature, compact myelin from donor mT-mNSC to be visualized in association with regenerated CST axon targets within the PLG bridge. We employed pharmacological immunosuppression for these allogeneic transplants because CST-GFP reporter mice were not on a constitutively immunodeficient background. While this resulted in reduced mT-mNSC survival and migration in comparison with transplantation into Rag-1 mice, we were able to identify mT-mNSC aligning with and ensheathing NF-H^+^ (Fig. 6L) and GFP^+^ CST (Fig. 6M) axons in the PLG bridge. Orthogonal projection clearly indicated that mT-mNSC fully surrounds GFP^+^ CST fibers (Fig. 6M1, blue arrowhead).

### The PLG bridge supports regeneration of a synaptic circuit connecting forelimb muscle to motor cortex.

An advantage of the PLG bridge implantation model is that axons can only reach the channels by regenerating; they cannot represent spared fibers. However, observation of axons in this region, even CST reporter axons, does not mean that these fibers have integrated into synaptic circuitry. We sought to test this key aspect of regeneration via transsynaptic PRV tracing. We injected GFP-reporter PRV virus into the forelimb triceps muscle ipsilateral to the SCI hemisection (Fig. 1C). PRV is retrogradely transported from the neuromuscular junction, sequentially infecting synaptically connected neurons to enable circuit tracing; we sought to use this method to identify whether neuronal wiring between injected muscle cells, spinal motor neurons, and sensorimotor cortical pyramidal neurons is re-established after PLG bridge implantation. Approximately 90% of CST axons originating from sensorimotor cortical pyramidal neurons decussate at the level of the pyramids. Thus, if CST motor axons have regenerated through the implanted PLG bridge and formed synaptic connections with spinal motor neurons, a majority of PRV labeling should be visualized in the contralateral motor cortex. In contrast, some brain nuclei (e.g. the paraventricular nucleus of the hypothalamus, PVN), would be expected to exhibit ipsilateral predominance of retrogradely labeled axons. PRV transsynaptic tracing was validated by injection into the triceps of 2-month-old naive (uninjured) mice, demonstrating a majority of labeling in contralateral motor cortex pyramidal neurons (Supplemental Fig. 4A, A2), sparse labeling in ipsilateral cortex pyramidal neurons (Supplemental Fig. 4A, A1), and predominant ipsilateral labeling of the PVN (Supplemental Fig. 4A, A3–4). These data are consistent with previous reports of PRV circuit tracing [54–58]. Because of the chronic nature of the hNSC transplants and analyses in these experiments, we further validated PRV tracing in this injection paradigm in 10-month-old naive mice (Supplemental Fig. 4B). Surprisingly, we found a reduction of retrograde PRV labeling in 10-month-old naive mice (Supplemental Fig. 4B, B1–3), suggesting that age impacts PRV labeling efficiency. This result could reflect a loss of initial viral infection efficiency at the muscle, a shift in the efficiency or timeline of retrograde viral transport, a weakening of synaptic connections in the spinal cord, or other factors [59].

**Figure 7:**
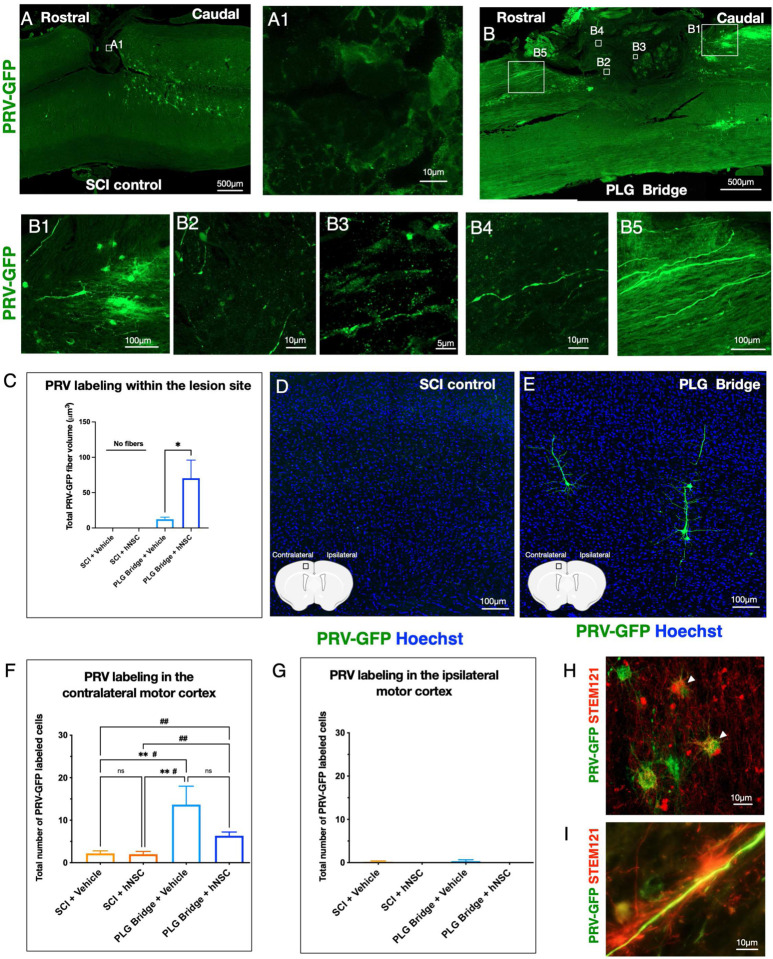
CST axons that regenerate through the PLG bridge are synaptically connected. **(A)** Horizontal section showing PRV labeling (green) in the control group; inset **(A1)** shows that PRV fibers were not observed in the SCI control lesion. **(B)** Horizontal section showing PRV labeling in the PLG group. Higher magnification insets show: PRV+ fibers caudal to the PLG bridge (**B1**); PRV^+^ regenerated fibers inside PLG bridge channels **(B2–4**); PRV^+^ fibers rostral to the PLG bridge (**B5**). (**C**) Quantification of PRV labeling in the lesion site. Statistical analysis by unpaired Student’s t-tests (*p<0.044; n = 3 biological replicates/group; Mean ± SEM). (**D-E**) Representative image of PRV labeling in the contralateral motor cortex. (**D**) SCI control and (**E**) PLG bridge groups. **(F-G)** Quantification of total PRV-labeled CST cell bodies in the motor cortex. **(F)** contralateral motor context and **(G)** ipsilateral motor cortex. (**H**) STEM121^+^ cells (red) stained positive for PRV (GFP, green), suggest stable integration into host circuitry. **(I)** Representative image of STEM121^+^ processes (red) warping around PRV fiber (green), suggest hNSC myelination of PRV labeled host axon. Groups were compared using one-way ANOVA, followed by Tukey post hoc tests (*P≤0.05, **P≤0.01). Statistical analysis by multiple unpaired t-tests. (#p≤0.05, ##p≤0.01; n = 3–5 biological replicates/group; Mean ± SEM).

We next assessed PRV transsynaptic tracing in lateral hemisected spinal cords within the lesion site as well as in the contralateral and ipsilateral motor cortex at 26 WPT (30WPI). At this experimental time point, the mice were ~10 months of age. No PRV labeling was detected in the lesion site of SCI control mice (Fig. 7A, A1). In contrast, robust PRV labeling was detected in regenerated axons within the channels of PLG bridge implanted mice (Fig. 7B, B2–4), as well as crossing the caudal-bridge and bridge-rostral tissue interface (Fig. 7B, B1 and B5). As observed for NF-H^+^ (Fig. 6G), comparison of Imaris volumetric data demonstrated that hNSC transplantation enhanced PRV^+^ axon regeneration (Fig. 7C). PRV labeling was also identified in choline acetyltransferase (ChAT) motor neurons (Supplemental Fig. 4D), but not calcitonin gene-related peptide (CGRP) labeled sensory fibers (Supplemental. Fig 4E), consistent with formation of a spinal motor neuron relay circuit. We next analyzed PRV labeling in the brain (Fig. 7F–H). The contralateral motor cortex exhibited clear evidence of PRV^+^ neurons in PLG bridge implanted vs. SCI control groups (Fig. 7D–F). While not significant, there was a trend for fewer PRV^+^ neurons in the PLG bridge + hNSC combination vs. PLG bridge + vehicle group (Figs. 7F). hNSC in the spinal cord were also PRV^+^, suggesting stable integration of donor human cells into mouse host circuitry (Fig. 7H), which could have diluted PRV transport to the brain because of an increased number of intermediate synaptic connections. PRV^+^ neurons were not observed in the ipsilateral motor cortex of PLG bridge or SCI control groups (Figs. 7G). The lesion model employed makes it unlikely that PRV labeling in the motor cortex could derive from spared motor neurons and/or CST crossing fibers innervating the triceps muscle above the level of the lesion. While the motor neuron pool innervating the forelimb extends from C2 through T1 in the rodent, motor neurons specifically innervating the triceps (the site of PRV injection) extend only from C5 through C8 [60]. Thus, the motor neurons innervating the triceps lie below the level of the C4/5 lesion site in our model. In this regard, the data shown in Fig. 7A, in which no PRV+ axons are observed within or above the level of the lesion in control animals, are consistent with a complete lesion of this connection. Similarly, it is unlikely that PRV labeling in the motor cortex represents a bypass of the lesion via the spared contralateral spinal cord and crossing fibers. We have shown previously that we do not observe the entry of axons from the contralateral spinal cord into the implanted bridge [14]; we replicated this in the Crym reporter mice employed in the present study. Thus, in this model, the contribution of any CST crossing fibers would have to take place below the level of the lesion. In this event: 1) PRV+ axons would be observed running in the spinal cord contralateral to the lesion/bridge; and 2) because over 90% of the CST decussates at the level of the pyramids, one would predict that a contralateral CST bypass would be observed primarily in the cortex ipsilateral to the lesion. However, PRV+ axons are absent in the spared tissue contralateral to the injury, and no PRV+ neurons were observed in motor cortex ipsilateral to the lesion. In sum, therefore, we interpret these data to demonstrate that motor cortex axons can regenerate through the PLG bridge and form synaptic connections with forelimb muscle, reconstituting motor circuitry. Moreover, we demonstrate novel evidence for the capacity of donor human NSC to similarly exhibit synaptic integration within a mouse host. Consistent with findings in Crym reporter mice (Fig. 6M), PRV^+^ axons exhibited close proximity to STEM121^+^ hNSC labeling, suggesting donor cell interaction and myelination of synaptically connected host axons (Fig. 7I).

### PLG bridge implantation and hNSC transplantation improve locomotor recovery.

**Figure 8:**
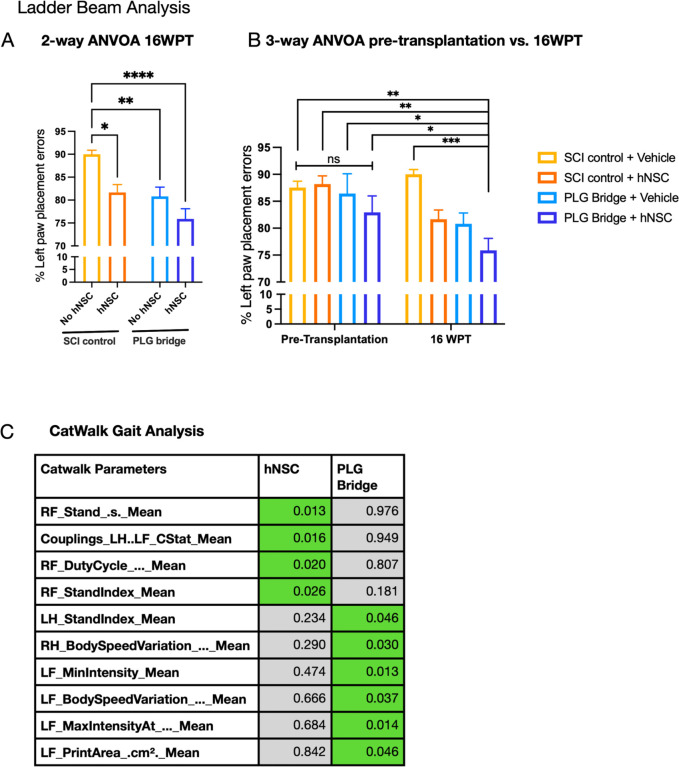
PLG bridge implantation and hNSC transplantation improve locomotor recovery in a synergistic manner. **(A)** Ladder beam data analysis shows that significant reduction in ipsilateral paw placement errors at 20 WPI (16 WPT) in the hNSC transplantation alone, PLG bridge implantation alone, and the combination of PLG bridge + hNSC groups. Statistical analysis via two-way ANOVA, followed by Tukey post hoc tests (*P≤0.05, **P≤0.01, and ****p ≤0.0001). Mean ± SEM; N= 9–12 animals per group. **(B)** 3-way ANOVA analysis of ladder beam data shows significant improvement in motor recovery only for the combination of PLG bridge implantation and hNSC transplantation. No significant differences in ladder beam performance were observed between the pre-transplantation (pre-T) groups. 3-way ANOVA, followed by Tukey post hoc tests (*P≤0.05, **P≤0.01, and ***p≤0.001). Mean ± SEM; N= 9–12 animals per group. **(C)** Unbiased multivariate analysis identifies a predominant effect of PLG bridge implantation on left (ipsilateral) limb function, and a predominant effect of hNSC transplantation on right (contralateral) limb function in CatWalk gait analysis. LF left forelimb; RF right forelimb; LH left hindlimb; RH right hindlimb. Table of p-values for two-way ANOVA analysis; N= 8–12 animals per group; CatWalk parameters highlighted in green have *p* value ≤ 0.05 for the effect of hNSC transplantation alone (Cells) vs. PLG bridge implantation alone (Bridge).

Mice were assessed for locomotor recovery at pre-injury, 4WPI (pre-transplantation), and 20WPI (16 WPT) using the horizontal ladder beam and CatWalk kinematic function tasks (Fig. 8) [14, 61–63]. We previously demonstrated that selective ablation of engrafted hNSC ablates gains in recovery of locomotor function [29], demonstrating a requirement for hNSC survival in repair. While we have shown that PLG bridge implantation post-SCI supports robust CST regeneration that is temporally linked to motor recovery [14], whether axonal regeneration is the underlying mechanism for repair has not been established. Therefore, we first used the same experimental SCI model, comparing SCI control, PLG bridge implantation, and retransection through the PLG bridge 10 weeks post-implantation. Catwalk kinematic analysis at 12 weeks post-implantation demonstrated that re-transection through the PLG bridge reverses locomotor performance to that of SCI control animals (Supplemental Fig. 5A-E), consistent with a requirement of regeneration through the bridge for recovery of function.

Additionally, we have previously reported that PLG bridge implantation and hNSC transplantation as separate interventions require 10–16 weeks to observe functional recovery [14, 29, 64, 65], consistent with a requirement for regeneration into and through the bridge, or donor cell migration/differentiation, respectively. Two-way ANOVA analysis of horizontal ladder beam errors at 16WPT with bridge (with/without) and cells (with/without) as variables demonstrated significant main effects for both PLG bridge implantation and hNSC transplantation; multiple comparison corrected t-tests also demonstrated a significant reduction in errors for PLG bridge alone, hNSC alone, and PLG bridge + hNSC groups (Fig. 8A). However, because hNSC were transplanted 30d after bridge implantation, we posited that further analysis could reveal the unique contribution of cell transplantation. To test the change in task performance after the addition of hNSC, horizontal ladder beam performance data collected pre-transplantation (4 WPI), was incorporated in a three-way ANOVA with time (pre-T vs. 16 WPT), bridge, and cells as variables, identifying significant main effects for each of these; however, multiple comparison corrected t-tests demonstrated that within this experimental window, only the combination of bridge + cells exhibited additional recovery of function compared to all pre-transplantation groups and the 16WPT control group (Fig. 8B).

We also conducted CatWalk kinematic gait analysis (Fig. 8C), applying an unbiased multivariate approach to test whether the effect of PLG bridge implantation on locomotion could be separated from that of hNSC transplantation on locomotion. This approach avoids *a priori* assumptions about which variables are meaningful to recovery. Univariate two-way ANOVAs were conducted in R using the “aov” (Analysis of Variance) function to analyze the independent effects of PLG bridge implantation and hNSC transplantation, identifying variables that had a p-value ≤ 0.05 (Fig. 8C). PLG bridge implantation and hNSC transplantation exhibited no overlap in the subset of variables identified in this analysis. hNSC transplantation altered four variables, three of which related to contralateral (right) forepaw movement. In contrast, PLG bridge implantation altered six variables, five of which related to ipsilateral (left) hind or forepaw movement. These data suggest parallel mechanisms on locomotor recovery, in which PLG bridge implantation exerts a predominant effect at the lesion, whereas hNSC transplantation exerts a predominant effect on the intact side of the spinal cord.

## DISCUSSION

Previous studies combining biomaterials and cell transplants have generally implanted seeded scaffold materials. Here, we investigated whether hNSC transplantation 30 days after an acutely implanted PLG bridge could migrate into the bridge channels when regenerated axons are located, enabling dissociation of the timing of these interventions. Consistent with our previous studies, transplanted hNSC migrated extensively in the spared parenchyma. PLG bridge implantation increased the total number of engrafted hNSC ipsilateral to the bridge for all three lineages vs. SCI control. This increase in ipsilateral engraftment may be due to increased mechanostability and tissue sparing adjacent to the injury after PLG bridge implantation, as we have reported previously [14]. It is also possible that the pro-regenerative environment driving axon regeneration into the PLG bridge also influenced hNSC engraftment. Most importantly, donor hNSC were preferentially associated with regenerating axons, migrating selectively within PLG bridge channels and distributing throughout the length of the bridge.

Donor NSC-derived oligodendrocytes contributed directly to myelination of mouse host axons, including corticospinal axons. This is the first report illustrating that donor NSC are capable of migrating along axons regenerating into a biomaterial scaffold and contributing to myelination of those axons. Moreover, we have previously shown that hNSC transplanted directly into the SCI epicenter become fate locked to generate astrocytes within and proximal to the lesion site [66]. In contrast, few human astrocytes were observed within or adjacent to the PLG bridge. These data indicate that multipotent hNSC that enter the PLG bridge follow a beneficial developmental program after transplantation, exhibiting the capacity to follow newly grown axons long distances and differentiation in a lineage appropriate manner.

PRV tracing demonstrated that CST axons that traversed the PLG bridge were synaptically connected to the ipsilateral forelimb muscle below the level of injury. This is the first report demonstrating the reestablishment of synaptic neural circuitry between the brain and neuromuscular junction after SCI via regeneration through a biomaterial bridge alone.

Previous studies have not demonstrated evidence of a synaptic circuit after biomaterial implantation without either cell transplantation into the lesion epicenter to create a bridge circuit, or pharmacological manipulation to lure axons into the caudal spared spinal cord [12, 67]. hNSC transplantation did not increase the number of PRV+ neurons detected in motor cortex. However, some hNSC-derived neurons in the spinal cord had integrated into mouse host spinal synaptic circuitry by 26 WPT. These data show that multipotent hNSC that enter the PLG bridge exhibit the potential to integrate with host circuitry via differentiation into not only myelinating oligodendrocytes, but also into synaptically connected neurons. Integration of hNSC into synaptic circuitry within the spinal cord could have affected the detection of synaptically connected motor cortex neurons by creating a viral ‘sink’. hNSC can also secrete factors that enhance endogenous regenerative mechanisms [50, 68–70], potentially diverting PRV labeling across other brain regions. This is supported by the observation that hNSC transplantation enhanced axon volume in the PLG bridge, as well as the observation that while hNSC do not generate Schwann cells, Schwann cell-derived myelination within the PLG bridge was increased in mice receiving hNSC transplants. While we were particularly interested in the CST and motor cortex because this projection is important for hand function in humans and refractory to regeneration, the integration of other brain areas could also contribute to repair. For example, brainstem motor pathways including reticulospinal and rubrospinal tracts play a major role in controlling rodent locomotion [71].

Achieving efficacy after hNSC transplantation in the chronic period post-SCI is widely considered to be a challenging goal. Here, we show that both acute bridge implantation and chronic hNSC transplantation impact functional locomotor recovery. Critically, statistical analysis factoring time (pre-T vs. 16 WPT), bridge, and cells as variables to focus on the contribution of hNSC transplantation on recovery of function, suggested an overall combinatorial effect of acute bridge implantation and chronic hNSC transplantation on recovery of function. Kinematic analysis suggested that motor recovery in the combination group is mediated in part through parallel mechanisms, in which PLG bridge implantation supported ipsilateral improvement whereas hNSC treatment supported contralateral improvement. In this context, hNSC are likely to exert pleiotropic mechanisms of repair that may include strengthening of spared motor circuity as well as enhancement of host axon regeneration and myelination of regenerated axons. In this regard, while behavioral data were collected at 16 WPT, identification of evidence for hNSC myelination of host axons in PLG bridge channels and hNSC integration into host circuitry by 24 WPT suggests that longer timepoints of analysis could be required to fully assess the impact of cell transplantation combined with PLG bridge implantation.

In sum, we demonstrate that hNSC transplanted in the chronic period after acute PLG bridge implantation are capable of migrating along regenerating axons into the PLG bridge channels, and enabling both donor cell-mediated myelination of regenerated host axons and donor cell integration into synaptic circuitry, along with gains in locomotor function. Alternative models, e.g. non-human primates, in which an extended time course of study is possible, may be necessary in order to reveal the full potential for this approach to spare tissue, promote regeneration, and maximize the function of new axonal connections. Regardless, these data identify a novel strategy that could open a new therapeutic window for SCI, particularly in cases of penetrating injury, where strategies that can enable true axonal regeneration may be required to open a path for recovery of function.

## MATERIALS & METHODS

### Ethics statements

Animal care, behavior acquisition, and data analysis were performed by investigators blinded to the experimental groups. All animal housing conditions, procedures, and animal care were approved by the UCI Institutional Animal Care and Use Committee (IACUC). Derivation and usage of human neural stem cell line UCI 161 for all *in vitro* and *in vivo* work was reviewed and approved by the UCI human Stem Cell Research Oversight Committee (hSCRO).

### Animal models

Profiling of the innate inflammatory microenvironment following SCI was performed using adult female C57BL/6 mice (JAXmice # 000664, The Jackson Laboratory, Bar Harbor, ME) (Fig. 2) and Rag1 mice (JAXmice # 002216, The Jackson Laboratory, Bar Harbor, ME) (Supplemental Fig. 1). hNSC transplantation studies were conducted in female immunodeficient Rag1 mice (Figs. 4–8). Multipotent cell membrane-localized tdTomato mouse neural stem cell (mT-mNSC) transplantation studies were conducted in male and female Crym-ZsGreen1 transgenic mice (Fig. 6L–M and Supplemental Fig. 3). Transgenic CRYM-ZsGreen1 mice were generated by crossing Crym^cre^ transgenic mice (MMRRC_036627-UCD) with Ai6 mice that contain a CAG promoter-driven enhanced green fluorescent protein variant (ZsGreen1) (JAXmice # 007906, The Jackson Laboratory, Bar Harbor, ME). Mu-Crystallin (Crym) is expressed in a diverse array of tissues, including layer V-VI of motor cortex in the brain. Cre recombinase-mediated excision of a floxed STOP enabled the expression of CrymCre: ZsGreen1 specifically in CST axons in the spinal cord; we have demonstrated previously that reporter expression is localized exclusively to CST axons in the spinal cord in these mice [14]. PLG bridge retransection study is performed in Transgenic EMX1:ROSA-CRYM-RFP mice. All mice were group housed with 2–4 cage mates.

### Spinal cord injury

Mouse C5 hemisection injury and postoperative care were performed as previously described [14]. Briefly, mice were anesthetized using 2% isoflurane for 5 minutes before surgery. After C5 laminectomy, mice received a unilateral hemisection at the left side with removal of a 1mm spinal cord segment followed by immediate implantation of PLG bridge. The laminectomy site was covered with sterile gelfoam (Pfizer, New York, NY) to prevent muscle adhesion to the PLG bridge. SCI control groups received only gelfoam at the lesion site. Rag1 mice were 11–19 weeks of age and CRYM-ZsGreen1 transgenic mice were 9–15 weeks of age at the time of injury. Mice were randomly allocated to different treatment groups. The exposed muscle was sutured using 5–0 chromic gut, and the skin was closed using wound clips. All mice were placed in cages on top of heating pads at 37°C overnight with clean Alpha-Dri bedding. The following subcutaneous injections were administered post-op: Baytril (2.5mg/kg) once a day for two weeks, lactated ringers (50mL/kg) once a day for five days, and buprenorphine (0.05mg/kg) every 12 hours for three days. Throughout the course of the study, bladder expression was performed manually twice per day. Since Rag1 mice are immunodeficient and CRYM-ZsGreen1 transgenic mice were on an immunosuppression paradigm, rotating antibiotics were given for the duration of the study to avoid bladder infections. Oral antibiotics dissolved in drinking water consisted of ciprofloxacin (10mg/100mL), Sulfamethoxazole/Trimethoprim (2mL/100mL), and/or ampicillin (20mg/mL) were each given for two weeks, and then rotated to the next antibiotic.

### Immune cell isolation post-injury and flow cytometric analysis.

Innate immune cell time course profiling following SCI was performed in C57BL/6 (JAX #000664, Bar Harbor ME) and Rag1 (JAX #002216, Bar Harbor ME) mice that received a C5 hemisection with bridge and gelfoam implantation or gelfoam only as above. C4-C6 vertebral dissection was performed 1 day post-injury (DPI), 1 week post-injury (WPI), 4WPI, 8WPI or 24WPI. Spinal cord tissue was dissociated using mechanical and enzymatic methods as previously described [72]. Myelin debris was removed with Myelin Removal Magnetic Beads II kit (Miltenyi Biotec, Auburn, CA) and an auto-MACS Pro Separator according to manufacturer instructions (Miltenyi Biotec, Auburn, CA). Cells collected after myelin removal were suspended in 0.85% ammonium chloride (diluted in sterile water) for five minutes to lyse red blood cells. The cell suspension was resuspended in PBS supplemented with 2% FBS (Thermo Fisher Scientific, Waltham, MA). Then the cells were stained with 7AAD viability dye (Thermo Fisher Scientific, Waltham, MA), CD45, CD11b, Ly6G, and CD68 antibodies as previously described [72]. The data was acquired using a BD FACS Aria II Flow cytometer (BD Biosciences, Franklin Lakes, New Jersey) and analyzed with FlowJo software. To ensure the detection of a true positive signal for each fluorescence channel, we used single stain compensation controls and fluorescence minus one (FMO) controls. Live cells (stained negative for 7AAD) were gated to analyze the number of CD45^+^CD11b^+^ myeloid events. The total myeloid cells were further gated to analyze the proportion of Ly6G^+^, Polymorphonuclear leukocytes (PMN), and CD68^+^ macrophages (MØ)/microglia subpopulations. Antibody sources, dilutions, and specificity are listed in Supplemental Table 1.

### Fabrication of multiple channel bridges for *in vivo* implantation studies

PLG bridges were generated using a gas foaming/particulate leaching method as previously described [17, 37]. The final bridge dimensions were 1.15 mm in length, 1.25 mm in width, and 2 mm in height and contained 9 channels.

### PLG scaffold fabrication for in-vitro hNSC differentiation assays

PLG pellets were comprised of a 75:25 lactide:glycolide ratio, with a molecular weight of 66,000–107,000 and an inherent viscosity of 0.55–0.75 (Sigma-Aldrich Inc, Saint Louis, MO). PLG pellets were placed in a silicone mold (8 × 9 × 1mm diameter) and melted at high temperature (130° C) on a hot plate. The liquid was compressed with a 1kg weight while the temperature was reduced 30°C every hour. Once the PLG cooled to room temperature, PLG scaffolds were peeled from the mold and sterilized for *in vitro* experiments with 70% ethanol, followed by one wash with sterile water and UV exposure in a laminar flow hood.

### hNSC line derivation and cell culture

Multipotent human neural stem cell line, UCI161 (referred to as hNSC) was established from fetal brain tissue procured at gestational 16 week and cultured as previously described [73]. Briefly, hNSC were fluorescence-activated cell sorting (FACS)-sorted to enrich for the CD133^+^ stem cell population. hNSC enriched for CD133 stem cell marker exhibit stable growth rate, sustained neurosphere-initiating capacity and multipotency *in vitro*, and differentiation into neural cell lineage cells and responsiveness to migration cues in a site-specific manner upon transplantation *in vivo* [30, 73]. hNSC were cultured as monolayers on poly-L-ornithine (PLO)/laminin (LAM) coated flasks in hNSC growth medium (GM). hNSC GM consists of X-VIVO 15 (Lonza, Basel, Switzerland) medium supplemented with 20 ng/mL bFGF (Invitrogen, Waltham, MA), 2 ng/mL EGF (Invitrogen, Waltham, MA), 2 ng/mL Heparin (Sigma-Aldrich Inc, Saint Louis, MO), 63 μg/mL N-acetylcysteine (NAC; Sigma-Aldrich Inc, Saint Louis, MO), 1x N2 supplement (Thermo Fisher Scientific, Waltham, MA), and 10 ng/mL LIF (Invitrogen, Waltham, MA).

### mT-mNSC Line Generation and Cell Culture

mT-mNSC line was established from cortices of 24Gt(ROSA)26Sortm4(ACTB-tdTomato-EGFP)Luo/J mouse (JAX mice #007576, The Jackson Laboratory, Bar Harbor, ME) at embryonic day 11.5 (E11.5) as previously described [30]. mT-mNSC ubiquitously expresses a membrane-targeted tdTomato reporter [21], which allows for visualization and identification of donor cell-derived myelin within the PLG bridge. mT-mNSC were cultured as a monolayer under the same growth conditions as hNSC. Similar to hNSC, mT-mNSC exhibits sustained neurosphere-initiating capacity in vitro, responsiveness to migration cues and the capacity to differentiate into three neural cell lineage cells upon transplantation in vivo. Both hNSC and mT-mNSC were fed twice a week and passaged every 1–2 weeks at 80% confluency.

### Generation of PMN or MØ conditioned media (CM) for hNSC *in vitro* fate analysis

To assess how immune cues impact hNSC after SCI in the Rag1 mice, we quantified the effect of innate immune cell conditioned media (CM) on hNSC fate *in vitro*. Polymorphonuclear leukocytes (PMN) and macrophages (MØ) were isolated from peritoneal cavities of adult female Rag1 immunodeficient mice, as previously described [74]. Briefly, mice were stimulated with 12% sodium caseinate (i.p. injection) and were sacrificed either 12–16 hours (to collect PMN) or 5 days (to collect MØ) post-injection (Fig. 1B and Supplemental Fig. 2). PMN and MØ (5,000,000 cells/ml) were cultured in hNSC differentiation media (DM). hNSC DM is X-VIVO 15 based media (Lonza, Basel, Switzerland) supplemented with 10 ng/mL GDNF (PeproTech, Rocky Hill, NJ), 10 ng/mL BDNF (PeproTech, Rocky Hill, NJ), 0.1 ng/mL bFGF, 10 μg/mL Ciprofloxacin (Cellgro), 2 ng/mL Heparin, 63 μg/mL NAC, and 1x N2 and 1x B27 supplements (Thermo Fisher Scientific, Waltham, MA). PMN CM was collected only once after 24hrs in culture. MØCM was collected once a day for three days, and the culture was replenished with fresh media after each collection. Both conditioned media were diluted (1:1) with fresh DM media and used for the hNSC differentiation experiments (UCI 161 cell line, passage 6).

### hNSC differentiation assays and fate analysis *in vitro*

To investigate the effect of PLG bridge on hNSC differentiation in presence of immune cues, hNSC were plated on either PLO/LAM coated PLG scaffolds or glass chamber slides (Fisher Scientific, Waltham, MA) and cultured for 14 days *in vitro* (DIV) with DM, PMN-CM, or MØ-CM. hNSC differentiated on glass or PLG scaffolds were fixed with 4% paraformaldehyde at room temperature for 20 minutes. hNSC were permeabilized and blocked with 0.1% Triton X-100 (Sigma-Aldrich Inc, Saint Louis, MO) and 10% donkey serum (Jackson ImmunoResearch, West Grove, PA) in PBS. Next, hNSC were immunostained for neuronal marker Tubulinß III (Tubß III), astrocytic marker glial fibrillary acidic protein (GFAP), and oligodendrocyte marker Olig2. hNSC were counterstained with nuclear dye Hoechst 33342 (1 μg/mL dilution; Invitrogen, Waltham, MA). Antibody sources and the dilutions were used as listed in Supplemental Table 1. Images were captured with 10X objective using random sampling with a ZEISS Axio Imager II light microscope with an Apotome2 image processor (Zeiss, Oberkochen, Germany). Imaris software (Oxford Instruments, Abingdon, United Kingdom) was used to quantify hNSC fate. Image acquisition and quantification were conducted by researchers that were blinded to the experimental groups. All experiments were conducted in biological triplicates or quadruplicates with a minimum of technical duplicates. Images for fate analysis were acquired and quantified with the same experimental parameters per substrate. Due to the high fluorescent background from the PLG scaffold, images were adjusted for brightness and contrast.

### Preincubation of PLG scaffold in MØ condition media and sequestration effect assessment

The sterile PLG scaffold was incubated in MØ-CM at 37° C for one week. Total protein concentration was quantified using a Pierce BCA Protein Assay Kit (Thermo Fisher Scientific, Waltham, MA) in control groups (MØ-CM) and MØ-CM + PLG scaffold preincubation (MØ-CM-PLG). Next, we assessed how PLG scaffold affects C1q concentration in both groups using a C1q ELISA kit (Hycult Biotech, Uden, The Netherlands) according to the manufacturer’s instructions.

### NSC transplantation *in vivo*

Four WPI, a second surgery was performed to transplant hNSC in Rag1 mice or mT-mNSC in CRYM-ZsGreen1 transgenic mice as previously described [29, 31, 44]. After re-exposure of the spinal cord at C5, cells were injected using a NanoInjector with micromanipulator (World Precision Instruments, Sarasota, FL) and siliconized beveled glass micropipettes (outer diameter = 100–110 mm; inner diameter = 70 mm; Sutter Instruments, Novato, CA). A total of 75,000 cells or vehicle (X-VIVO media) in 1uL total volume was delivered to the spared spinal parenchyma at four sites, two rostral and two caudal to the lesion, 250nL per site (schematic in Fig. 1C). All mice received post-operative care as described above.

### Immune suppression in CRYM-ZsGreen1 transgenic mice:

Mouse-to-mouse allogeneic transplantation requires a pharmacological immunosuppression protocol to enable survival of transplanted mNSC within the host. To establish our immunosuppression protocol, we tested combined treatment with both Cyclosporin A (CsA; Perrigo, Minneapolis, MN) and anti-CD4 (Invitrogen, Waltham) in C57Bl/6 mice that received a C5 hemisection SCI. The final immunosuppression regimen was as follows: 10mg/kg s.c. CsA 2 days prior to mNSC transplantation continued daily until sacrifice, combined with 5mg/kg i.p. anti-CD4 1 day prior to mNSC transplantation, continued bi-weekly until sacrifice.

### Pseudorabies Virus (PRV) tracing

PRV-152-GFP trans-synaptic tracer (2.48 X 109 pfu/ml; in collaboration with Dr. Xiangmin Xu, UCI) was injected into the ipsilateral (left) forelimb triceps muscle of the animals at 30 WPI. This allowed for retrograde labeling of cortical pyramidal neurons and validation of functional connectivity after PLG bridge implantation. Following anesthesia (1.5% isoflurane in O_2_ for 10 minutes), a skin incision was made to expose the left triceps forelimb muscle. Using a stereotaxic Hamilton syringe with a 26-gauge needle, 5 μL PRV was injected into two sites of the left triceps forelimb muscle at different depths with a five-minute wait period between each injection. Skin was closed using tissue adhesive (Vetbond, St. Paul, Minnesota, USA). Animals received subcutaneous injections Carprofen (5mg/kg) injection and bladder care twice a day. Four days post-PRV injection, mice were perfused, and the spinal cord segment (C3 to C7) and brain tissues were dissected for further analysis.

### Perfusion, Tissue Collection, and Sectioning

Mice were anesthetized using pentobarbital (100 mg/kg) and transcardially perfused with PBS (15 mL) followed by 4% paraformaldehyde (100 mL) at 16 weeks post-transplantation (WPT) (hNSC differentiation fate and regeneration assessment) or 26 WPT (PRV connectivity tracing). Brain tissue and spinal cord segments corresponding to C3-C7 roots were dissected and post-fixed/cryoprotected overnight in 4% paraformaldehyde + 20% sucrose. The tissue was then flash frozen using −60°C isopentane and stored at 80°C. Brain tissues were transversely sectioned using a sliding microtome, and 30μm serial sections were collected in phosphate-buffered saline with 0.02% sodium azide and stored at 4°C until further use. C2-C8 spinal cord segments were embedded in Neg50 Frozen Section Medium (Fisher Scientific International, Hampton, NH) to be processed into 30 μm thick sections with a cryostat and Cryo-Jane tape transfer system (Leica Biosystems, Wetzlar, Germany). Spinal cords were sectioned either transversely or horizontally, and the slides were stored at −20°C until processed for immunohistochemistry.

### Immunohistochemistry and imaging

Slides with spinal cord tissue sections (CryoJane tape transfer slides) were incubated at 60°C on a hot plate for 2 hours, dipped in Histo-Clear II clearing agent (National Diagnostics, Atlanta, Georgia) for 20 minutes, rehydrated in a descending ethanol gradient (100%, 95%, 80%, and 70%) 5 minutes in each solution, and hydrated in distilled water for 10 minutes. Then for antigen retrieval, slides were immersed in a preheated sodium citrate antigen retrieval solution (10 mM sodium citrate, 0.05% Tween 20, pH 6.0) for 30 min at 96°C in a water bath. Following a 20-minute cool down at room temperature, the slides were rinsed in water and processed for immunohistochemistry as described below. Spinal cord sections (CryoJane tape transfer slides) and brain sections (microtome, free-floating) were incubated with blocking buffer (1.5% donkey serum and 0.1% Triton X in PBS) for an hour at room temperature and then incubated with primary antibody (diluted in blocking solution) overnight at room temperature. After 3 five-minute washes (0.1% Triton-X 100 in PBS), tissue was incubated with appropriate fluorescent-dye conjugated secondary antibodies and nuclear dye Hoechst 33342 (1 μg/mL dilution; Invitrogen, Waltham, MA) for 2 hours at room temperature. Following 3 final five-minute washes (0.1% Triton-X 100 in PBS), slides were mounted with Fluoromount G (SouthernBiotech, Birmingham, AL). Images were captured using a ZEISS Axio Imager II light microscope with an Apotome2 image processor or a ZEISS LSM 900 with Airyscan 2 microscope (Zeiss, Oberkochen, Germany).

### hNSC fate quantification *in vivo*

For hNSC fate quantification, 16 WPT transverse tissue sections (1/12 sampling interval) were processed for immunohistochemistry in the following sets: STEM121^+^ (human cells) only, STEM121^+^/Olig2^+^ (human oligodendrocytes), STEM121^+^/DCX^+^ (human neuronal precursors) and STEM121^+^/GFAP^+^ (human astrocytes). An ApoTome microscope (Zeiss) microscope system and 20x objective were used to capture 30–40 μm Z-stacks of optical slices in 2 μm intervals. Migration of human cells in the rostral and caudal parenchyma was determined with unbiased stereology using systematic random sampling, an optical fractionator probe, and Stereo Investigator (version 2020.1.3, MicroBrightField, Williston, VT). Although hNSC migration was observed throughout the isolated spinal cord section (collected from C2-C8 segments), stereological quantification of hNSC in the spared parenchyma was performed within a range of 2160 μm rostral and 2160μm caudal to the lesion site (approximately C3-C7). Parameters for analysis were: grid size of 450 μm × 450 μm, frame size of 70 μm × 70 μm, and guard zone of 2 μm. Probe grid size and counting frame size were empirically determined to yield average cumulative error values <0.1. In contrast, quantification at the lesion site was performed in single sections, because: 1) hNSC distribution was not homogeneous within the bridge, localizing to the channels; 2) the number of sections was limited because of the small lesion size and post-fixation shrinkage, yielding only ~10 total sections through the bridge per mouse. Within the bridge, all hNSC were manually quantified using StereoInvestigator. Within the spared tissue contralateral to the lesion, hNSC were quantified using systematic random sampling with StereoInvestigator.

### Axonal regeneration and myelination quantification

To assess total regenerated and myelinated neurofilaments within the SCI lesion site and PLG bridge, we used transverse sections from spinal cords collected at 16 WPT. Since the number of spinal cord sections with lesion was limited, as noted above, analysis was performed on one section per animal. Triple immunostaining was performed using NF-H (Neurofilament Heavy Chain, axonal marker), MBP (Myelin Basic Protein, pan myelination marker), and P0 (Myelin Protein Zero, Schwann cell myelin). Antibody sources and dilutions used are listed in Supplemental Table 1. For quantification, four to six random optical fields within the lesion per section were imaged using a Zeiss LSM 900 with Airyscan super resolution microscope. For each optical field, 6 μm Z stack images (0.3 μm Z-step) were captured using a 60X oil objective. 3D surface volume rendering was performed using Imaris v9.6 (Oxford Instruments, Abingdon, United Kingdom). Briefly, all three volumes of total neurofilament, total MBP positive myelinated neurofilament, and total P0 positive myelinated neurofilament were masked using the Surface feature. To exclude excess noise, a filter was made to a minimum voxel size of around 1000 for each image. when masking the surface volumes. Next, the NF-H positive surface volume of neurofilament that was associated with the oligodendrocyte-derived myelin (MBP^+^PO^−^ volume) and Schwann cell-derived myelin (MBP^+^P0^+^ and MBP^−^P0^+^) was masked using the object-to-object shortest distance (0.4 um) filter in Imaris software.

### PRV-GFP labeling quantification

Spinal cord tissue for these analyses was collected at 30 WPI and sectioned horizontally at 30μm thickness using CryoJane tape transfer method. Immunostaining for ⅙ sampling interval sections was performed to detect PRV-GFP labeled fibers. For quantification, five to six random optical fields within the lesion site per section were imaged using Zeiss LSM 900 with Airyscan super resolution microscope. Antibody source and the dilutions were used as listed in Supplemental Table 1. For each optical field, 24 μm Z stack images (0.4 μm Z-step) were captured using 60X oil objective. PRV-GFP labeled filament volumes were manually traced using Imaris v9.6 filament manual tracing software (Oxford Instruments, Abingdon, United Kingdom). Brain sections were sectioned coronally at 30μm using a sliding microtome. Immunostaining (all sections containing motor cortex, no sampling) was performed to detect PRV-GFP cell bodies. All PRV^+^ cell bodies within the motor cortex were counted manually. Quantification was performed at 20X magnification using ZEISS Axio Imager II light microscope with an Apotome2 image processor. Image acquisition and quantifications were performed by investigators blinded to the experimental groups.

### Behavioral Testing and Locomotor Recovery Assessment

All behavioral data were collected and analyzed by observers blinded to the experimental groups. Ns as indicated under statistical analysis and exclusions below. Mice were handled daily for two weeks prior to pre-injury behavioral testing to acclimate animals to human contact. Horizontal ladder beam and CatWalk Gait Acquisition were performed to quantify changes in motor recovery and kinetic parameters after injury. For Rag1 mice, locomotor assessments were acquired pre-injury, pre-transplantation, and 16 WPT timepoints. *Horizontal ladder beam*: Following a five-minute acclimation to the environment, mice were recorded walking across a horizontal ladder beam for three trials. Video analysis quantified each left paw placement error, as previously described [61]. *CatWalk Gait Acquisition*: Following a five-minute acclimation, each animal performed three runs across the LED lighted glass stage of CatWalk XT (Noldus Information Technology Inc., Leesburg, VA). A successful run contained ten consecutive steps, a consistent walking speed, and no paw placement on the stage walls. Runs were auto-classified using the CatWalk software version 10.1 [75]. For the bridge retransection study Catwalk data analysis was performed and analyzed at 12 WPI. For analysis, we utilized Gradient Booster Machine (GBM), a decision tree algorithm, implemented in R package (“gbm” version 2.1.8.1) analysis [76], which has been successfully applied to Catwalk measurements [77] and was used to select a subset of the most informative variables to predict the therapeutic outcomes. Consequently, fifteen variables of the original Catwalk data were used as input to a linear discriminate (LD) function implemented in R package (“klaR, version 1.7–2) [78] to define the linear boundary that separates the treatment groups in the reduced feature space. The outcome result was visualized by using ggcord/ggplot2 R package (version 3.4.1) [79] and the LD weights were retrieved for each variable.

### Statistical analysis and exclusions

Statistical analysis of data was conducted as indicated in the figure legend for each figure. Mice were randomized across groups to ensure an unbiased distribution by age and weight by an investigator not involved directly in the study. A total of 17 Rag-1 mice in the main transplantation study were excluded using pre-hoc criteria by an investigator blinded to the experimental groups and not involved directly in the study. Exclusion criteria: excessive weight loss, autophagy, staph infection of the hair follicles, or surgical complication (n =12); anatomical defect (n =2); failed bridge apposition (n = 1); Exclusion criteria: excessive weight loss, autophagy, staph infection of the hair follicles, or surgical complication (n =12); anatomical defect (n =2); failed bridge apposition (n = 1); failed transplantation/engraftment (n =1); and Grubbs’ test for outliers (n=2). Final Ns for behavioral analysis were as follows. Ladder beam: SCI control + Vehicle (n=10), SCI control + hNSC (n=10), PLG bridge + Vehicle (n=9), and PLG bridge + hNSC (n=12). Catwalk analysis: SCI control + Vehicle (n=8), SCI control + hNSC (n=9), PLG bridge + Vehicle (n=9), and PLG bridge + hNSC (n=12). For Catwalk, two mice in SCI control + Vehicle group and one mouse in the SCI control + hNSC group could not perform the task and were excluded from statistical analysis for this task but were retained for horizontal ladder beam analysis and histology.

## Data Availability

The data that support the findings of this study are available from the corresponding author upon reasonable request.
